# Simultaneous Integrated Boost Intensity-Modulated Radiotherapy for Locally Advanced Drug-Resistant Gastrointestinal Stromal Tumors: A Feasibility Study

**DOI:** 10.3389/fonc.2020.545892

**Published:** 2020-11-23

**Authors:** Longhao Li, Xin Yi, Haixia Cui, Xuemei Zhao, Jun Dang, Qingfeng Jiang, Ying Li

**Affiliations:** ^1^ Department of Oncology, The First Affiliated Hospital of Chongqing Medical University, Chongqing, China; ^2^ Department of Oncology, The Dazu District People’s Hospital, Chongqing, China

**Keywords:** gastrointestinal stromal tumors, locally advanced, drug-resistant, simultaneous integrated boost, intensity-modulated radiation therapy

## Abstract

**Background:**

As an emerging clinical problem, locally advanced drug-resistant gastrointestinal stromal tumors (LADRGISTs) has relatively few therapeutic schemes. Although radiotherapy is not often considered for GISTs, it could be a valuable contributing modality. The aim of our study is to explore a safe and effective radiation regimen for LADR-GISTs.

**Methods:**

Three patients with LADR-GISTs were treated with simultaneous integrated boost intensity-modulated radiation therapy (SIB-IMRT) plans. In the SIB-IMRT plans, gross target volume (GTV) was divided into GTV-outer, GTV-mid, and GTV-center. And the prescribed dose of planning gross target volume (PGTV) and GTV-outer were both set to 50.4 Gy in 28 fractions. GTV-mid and GTV-center were simultaneously boosted to 60–62 Gy and 62–64 Gy respectively. For comparison purposes, conventional IMRT (Con-IMRT) plans with uniform dose distribution were generated for same optimization objectives without a dose boost to GTV-mid and GTV-center. All plans were optimized to make sure that deliver at least 95% of the prescription dose was delivered to PGTV. Isodose distribution, dose profiles, conformity indexes (CIs), monitor units (MUs), and dose volume histogram (DVH) was evaluated for each individual patient. After the three patients were treated with SIB-IMRT plans, the relative changes in the tumor size and CT values by CT scanning were also tracked.

**Results:**

Compared with Con-IMRT plans, SIB-IMRT plans saw a significant increase from D_95_ to D_2_ of the GTV. With steeper dose gradients in the dose profiles, SIB-IMRT plans had GTV-mid and GTV-center accumulated with higher dose mainly by delivering extra 93 MUs in average. However, there was no significant difference in CIs and organs at risks (OARs) DVH. The relative changes in tumor size and CT values of the three patients in follow up were up to the Choi criteria and the three patients were all assessed as partial response.

**Conclusions:**

The proposed SIB-IMRT may be a potential technique for achieving objective response and prolonging survival of selected GISTs patients.

## Introduction

Gastrointestinal stromal tumors (GISTs) are the most common mesenchymal neoplasm of the gastrointestinal tract, arising from the interstitial cells of Cajal. The relevant researches reported the pathogenesis of GISTs are mainly related to mutations in the tyrosine kinase receptor (KIT) and/or platelet-derived growth factor receptor alpha (PDGFRA) gene (1–3). Currently, GISTs are typically treated with resection and adjuvant therapy with tyrosine kinase inhibitors (TKIs) at high risk for recurrence (4, 5). Unresectable or metastatic tumors are treated primarily by TKIs therapy (6, 7). Under the current treatment guidelines, radiotherapy is not a recommended option, or is only used for palliative intent of bone metastases (8).

Historically, GISTs have been considered to be relatively insensitive to radiotherapy, just as most other soft issue sarcomas. So far, a significant number of publications have demonstrated that GISTs are not uniformly radioresistant and radiotherapy could be beneficial to the management of GISTs. Pollock et al. (9) presented that a patient who underwent 50.4 Gy postoperative radiation after a R1 resection of a 7-cm rectal GIST, did not relapse with two years. Ciresra et al. (10) reported that radiotherapy combined with TKIs therapy resulted in a lesion reduction in a case of rectal GIST. They concluded that a pathologic complete response can be achieved with a dose of 50.4 Gy. Subsequently, a number of case reports provided insight into the efficacy of radiotherapy (11–13). In a retrospective series of 15 patients, Cuaron et al. (14) suggested that GISTs were more sensitive to a higher radiation dose. After reviewing the literature of radiotherapy in rectal GISTs, Ozkan (15) demonstrated that GISTs were radiosensitive in long-term local control and most patients could benefit from radiotherapy with palliative, adjuvant or definitive intent. According to the above, in certain circumstances, GISTs are radiosensitive and radiotherapy can be a valuable alternative in GISTs management.

According to the previously mentioned, radiotherapy could be regarded as a promising and viable option for GISTs. However, radiotherapy for GISTs was still limited to dose-limiting toxicity of the adjacent small bowel. In recent years, intensity-modulated radiation therapy (IMRT), image-guided radiation therapy (IGRT) and other technological advances has realized dose escalation in target volume and potential reduction in acute and delayed toxicity by facilitating treatment delivery and normal tissue protection (6). Moreover, simultaneous integrated boost intensity-modulated radiation therapy (SIB-IMRT) can deliver the highest possible dose to target volume and increase tumor response without significant increase of healthy tissue irradiation (16, 17). This technique has been successfully applied to the several types of bulky tumors, such as esophageal cancer, head and neck tumors, lung cancers, pelvic tumors, and soft tissue sarcomas (18–22). A better biochemical control can be achieved in SIB-IMRT by increasing dose (23). Therefore, SIB-IMRT may offer a valuable alternative option for patients of moderately radiosensitive GISTs.

In current clinic practice, locally advanced drug-resistant GISTs (LADR-GISTs) that are technically unresectable and failed in systemic TKIs therapies have emerged as a common clinical problem, with relatively few therapeutic schemes. To the best of our knowledge, there is no report about SIB-IMRT for LADR-GISTs. In this study, we designed a novel SIB-IMRT plan and the dose was gradually escalated from the peripheral region of GTV to the center region. The focus of this study was to compared efficacy and toxicity between conventional IMRT (Con-IMRT) plans and SIB-IMRT plans and explore a safe and practical radiation regimen for LADR-GISTs.

## Methods and Materials

### Patient, Tumor, and Treatment Characteristics

From 2016 to 2019, three patients with LADR-GISTs were treated with SIB-IMRT. The enrolled patients are 62, 50, and 56 years old at diagnosis. They underwent R_0_ resection of the primary tumor as soon as the disease was detected, and then started on systemic TKIs therapies. After a period of time (median time: 3 years), their tumors recurred due to drug resistance. Moreover, the progression of lesions were detected in all enrolled patients. Due to the resistance to TKIs therapy and lack of surgical options, they received radiation therapies to relieve symptoms (such as poor appetite, bloating, abdominal pain, frequent urination and constipation). **Table 1** illustrates the summary of patients, tumors, and treatment characteristics in detail.

**Table 1:** Patient, tumor, and treatment characteristics.

No.	Age (diagnosis/RT)	Primary tumor site	Initial tumor size	Type of resection	TKIs therapy	Indication for RT	Tumor size before RT	RT site
1	62/67	Small intestine	10cm	R_0_	Imatinib/sunitinib	Progression on TKIs resistance and unresectable	18.0cm	Abdomen and pelvic
2	50/55	Ileum	4.3cm	R_0_	Imatinib/sunitinib	Progression on TKIs resistance and unresectable	17.2cm	Abdomen and pelvic
3	56/60	Jejunum	15cm	R_0_	Imatinib/sunitinib	Progression on TKIs resistance and unresectable	20.0cm	Abdomen and pelvic

*No.: patient number; initial tumor size: the maximum diameter of tumor in CT imaging; R0: no residue under the microscope after surgical. Tumor size before RT: the maximum diameter of tumor in CT imaging; abdomen and pelvic: abdomen and peritoneal seeding mass in pelvic.

### Clinical CT Data and Volume Definition

**Figure 1:**
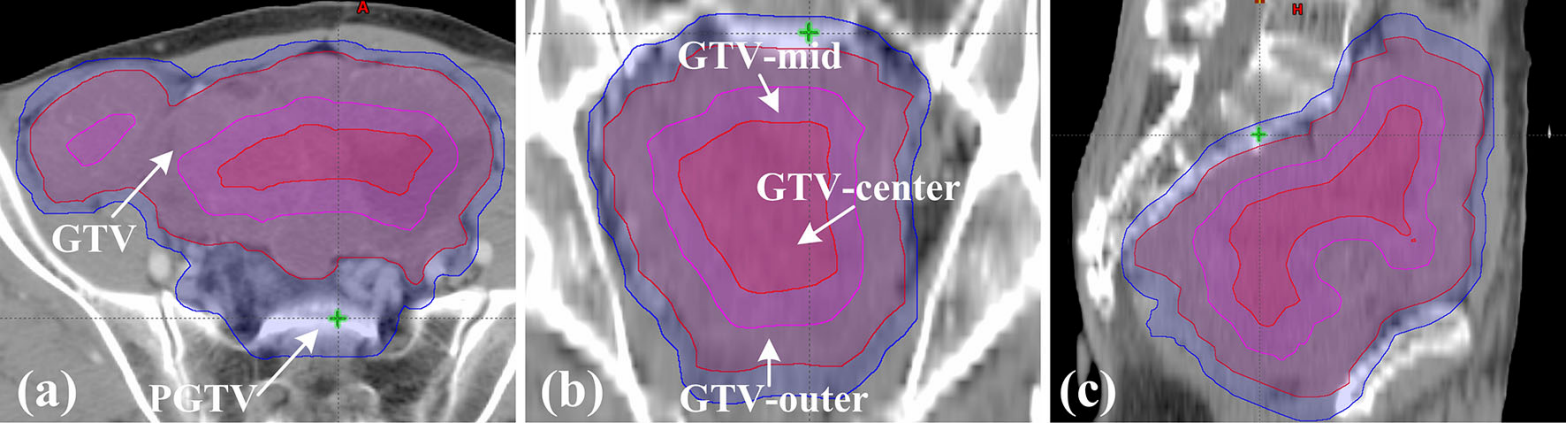
Definition of target volume in patient 1. **(A)** The axial plane; **(B)** the coronal plane; **(C)** the sagittal plane.

Each patient underwent computed tomography simulation in the supine position using GE CT scanner (GE Medical Systems, Milwaukee, WI). The CT scan covered the total abdomen and pelvic cavity. Moreover, all patients were instructed to drink 400 ml of water and empty the rectum in one hour prior to the CT scan, and they were advised to follow the same instructions in daily radiotherapy. The gross tumor was defined as GTV. Due to a rare (1-2%) lymph-node metastases in GISTs, the lymphatic drainage of the gross tumor were not irradiated as the clinical target volume (CTV) (24–26). The planning gross target volume (PGTV) was obtained by applying an isocentric margin of 5mm to the GTV. OARs mainly contained the rectum, bladder, and intestines. The rectum ranged from the anus to the junction of the rectal sigmoid colon. Due to the squeezing action of large tumor, it was difficult to distinguish the small bowel and the large intestine. Thus, both of them were included in the intestines in our research. The normal tissue (NT) structure was Body minus PGTV. In addition, GTV was divided into GTV-outer, GTV-mid and GTV-center as required by SIB-IMRT. As shown in **Figure 1**, these structures were detailed in the three standard orthogonal planes. GTV-center was created with an isocentric contraction of 2–3 cm in GTV. GTV-mid was defined as the GTV minus 1-2 cm excluding GTV-center. The rest of GTV was defined as GTV-outer. The contraction in GTV-mid and GTV-center was determined by the relative location between OARs and GTV in order to avoid hotspots in the overlap regions of OARs and GTV. All these structures were contoured with Eclipse v. 13.5 (Varian Medical Systems, Palo Alto, CA) by an experienced oncologist, and were reviewed by another senior oncologist.

### Treatment Planning

In this study, Con-IMRT plan and SIB-IMRT plan were offered to each patient. In SIB-IMRT plan, GTV was divided into three parts (GTV-outer, GTV-mid, and GTV-center) to obtain inhomogeneity dose in target volume. Therefore, the prescribed dose of PGTV and GTV-outer were both set to 50.4 Gy in 28 fractions. However, the presibribed dose of GTV-mid and GTV-center were boosted to 60–62 and 62–64Gy respectively. The detailed dose objectives of different patients were listed in **Table 2**. For comparison purposes, the Con-IMRT plans with PGTV and GTV being only set to 50.4 Gy were generated in the same beam arrangement. The optimization objectives of specific structure in Con-IMRT plan for each patient were same with that in SIB-IMRT plan except for GTV. All plans were calculated on a 2.5mm isotropic dose grid with anisotropic analytical algorithm (AAA) through Eclipse v.13.5 (Varian Medical Systems, Palo Alto. CA. USA). They were performed with six MV photon beams from a Varian-21EX linear accelerator. Dynamic MLC delivery (sliding window) was selected as the delivery method. In addition, all plans were made by an experienced medical physicist and reviewed by a senior medical physicist.

**Table 2:** Dose objectives of gross target volume (GTV) for simultaneous integrated boost intensity-modulated radiation therapy (SIB-IMRT) plans and conventional IMRT (Con-IMRT) plans in three patients.

Category	Structure	Dose objectives (Gy)		
		Patient 1	Patient 2	Patient 3
SIB-IMRT	GTV-outer	D_100_≥50.4, D1cc ≤ 56	D_100_≥50.4, D_1cc_ ≤ 56	D_100_≥50.4, D_1cc_ ≤ 56
	GTV-mid	D_100_≥60, D1cc ≤ 62	D_100_≥60, D_1cc_ ≤ 62	D_100_≥60, D_1cc_ ≤ 62
	GTV-center	D_100_≥62	D_100_≥64.4	D_100_≥64.4
Con-IMRT	GTV	D_100_≥50.4, D_1cc_ ≤ 56	D_100_≥50.4, D_1cc_ ≤ 56	D_100_≥50.4, D_1cc_ ≤ 56

### Plan Analysis and Evaluation

**Figure 2:**
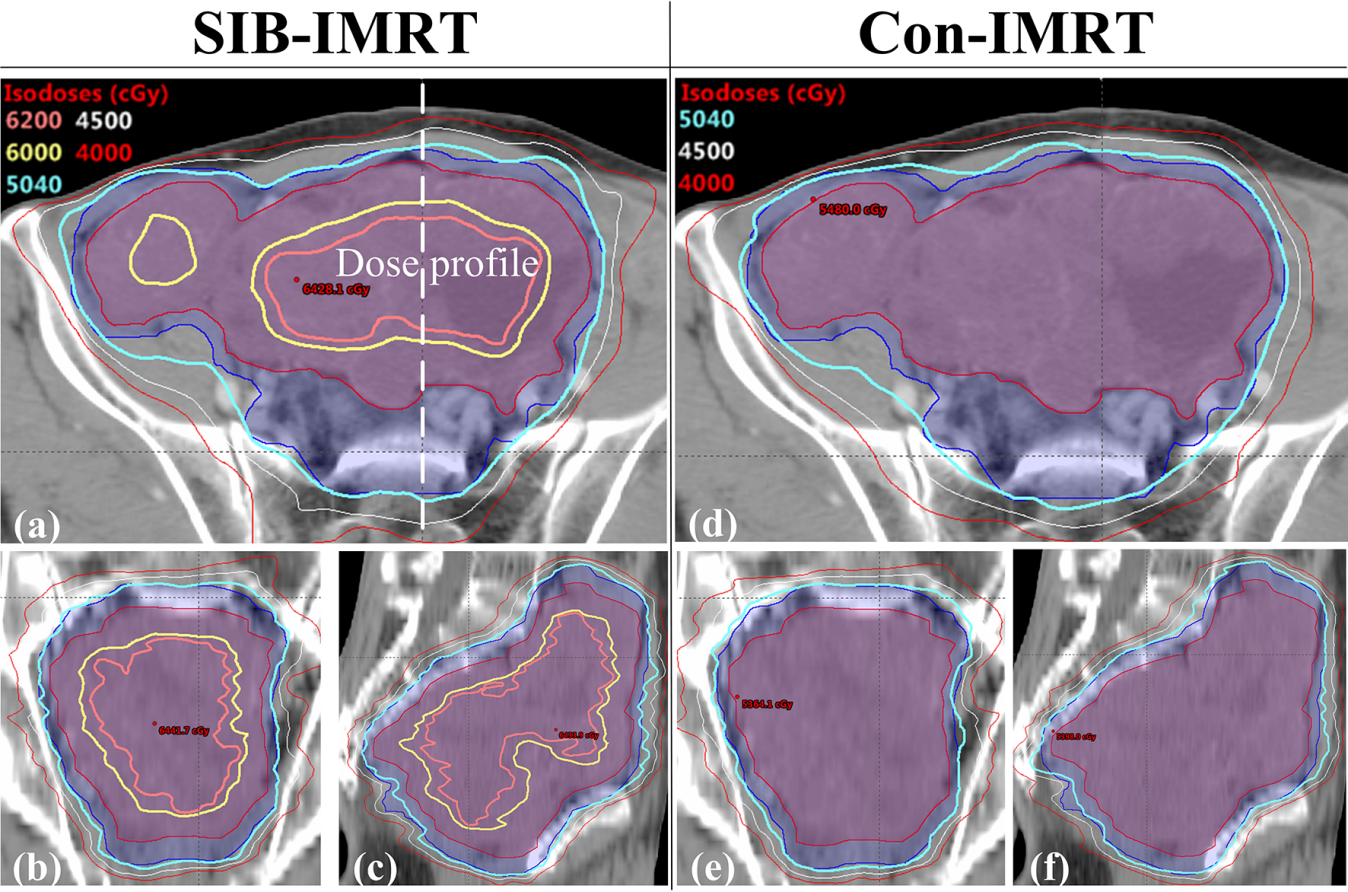
Comparison of the isodose distribution in patient 1. **(A)** the axial plane in the SIB-IMRT plan; **(B)** the coronal plane in the SIB-IMRT plan; **(C)** the sagittal plane in the SIB-IMRT plan; **(D)** the axial plane in the Con-IMRT plan; **(E)** the coronal plane in the Con-IMRT plan; **(F)** the sagittal plane in the SIB-IMRT plan.

To perform a better analysis and evaluation, both Con-IMRT plans and SIB-IMRT plans were normalized by having 95% of the PGTV receive 100% of the prescribed dose. Dose-volume histograms (DVH) was applied for calculation and evaluation of GTV, PTV and OARs. The dose profile (along the dashed line drawn in **Figure 2A**, PGTV’s conformality indexes (CIs: ratio of total volume receiving 95% of prescription dose to planning target volume receiving 95% of prescription dose) and moniter units (MUs) were obtained for comparison (27). In addition, DVH information of OARs, such as V_20_, V_30_, V_40_, V_45_, V_50_, D_1cc_, and D_2cc_, was also compared.

### Treatment and Follow-Up

All patients received the treatment of SIB-IMRT plans. In order to minimize the influence of structure movement, they were advised to they were advised to keep their bladder full and rectum empty during every radiation therapy. Daily cone-beam CT imaging was carried out before daily radiotherapy. CT scanning were provided for all patients in 3 months after final treatment and every 6 months thereafter. In order to perform quantitative evaluation to the response of irradiated lesions, the tumor size of three patients was measured by the maximum diameter in three planes (the axial, coronal, and sagittal plane). Meantime, the corresponding CT values was extracted from the same area with abundant blood supply at the arterial phase. Tumor response to radiotherapy was assessed by Choi criteria. Choi criteria includes the following four response categories: complete response (CR: Disappearance of all target lesions), partial response (PR: Decrease in tumor size ≥10% or decrease in tumor density ≥15% on CT), stable disease (SD: Does not meet the criteria for CR, PR or PD) and progressive disease (PD: Increase in tumor size ≥10% and does not meet PR criteria by tumor density).

## Results

### Results of Plan Evaluation

**Figure 3:**
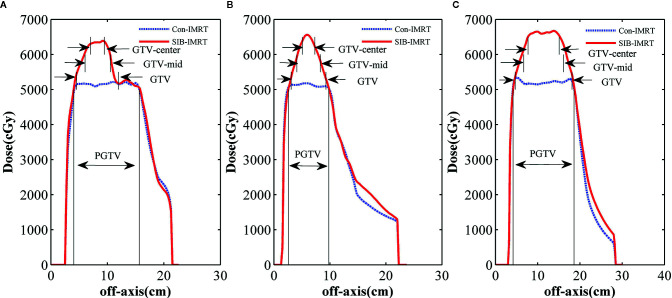
Profile comparisons in three patients. **(A)** Patient 1; **(B)** patient 2; **(C)** patient 3.

**Figure 2** shows the isodose distribution comparison of patient 1. The coverage of 4,000 cGy isodose in SIB-IMRT plan was largely consistent with that in Con-IMRT plan. However, the escalating isodose of SIB-IMRT plans in GTV was clearly identifiable in three orthogonal planes. And the GTV-center received a dose in excess of 62Gy (123% of the prescribed dose in PGTV). As shown in **Figure 3**, the dose profile comparison of three patients clearly demonstrated the steeper dose gradients within the GTV for SIB-IMRT plans. It was worth noting that a higher dose was mainly concentrated in GTV-mid and GTV-center. In addition, **Figure 3** shows that the profiles of SIB-IMRT plans excluding PGTV are nearly consistent with that of Con-IMRT plans. The CIs and MUs are shown in **Table 3**. Along with higher boost dose in GTV, the total MUs of SIB-IMRT plans are 93 MUs higher than that of Con-IMRT plans in average. Nonetheless, there is little difference in the CIs between the two plans of each patient.

**Table 3:** Comparisons in conformity indexes (CIs) and monitor units (MUs) between conventional intensity-modulated radiation therapy (Con-IMRT) plans and simultaneous integrated boost-IMRT (SIB-IMRT) plans.

Patient No.	Group	CIs	MUs
1	Con-IMRT	0.899	645
	SIB-IMRT	0.901	763
2	Con-IMRT	0.927	326
	SIB-IMRT	0.932	370
3	Con-IMRT	0.911	724
	SIB-IMRT	0.912	843

**Figure 4:**
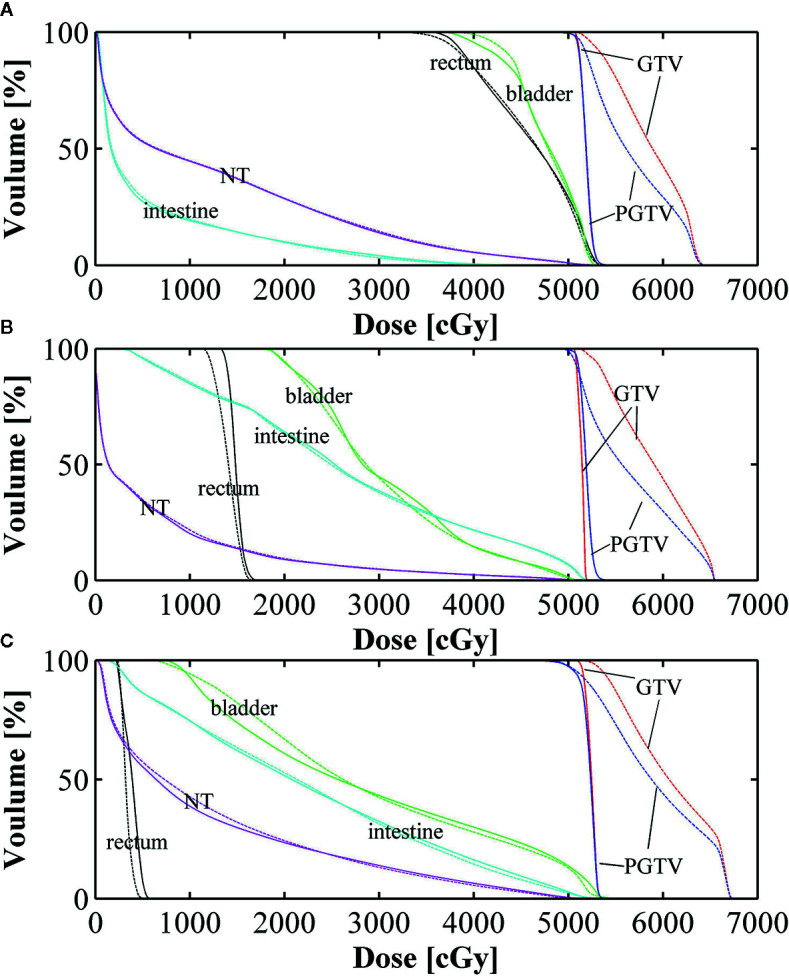
Dose volume histogram (DVH) comparisons between conventional intensity-modulated radiation therapy (Con-IMRT) (solid line) and simultaneous integrated boost-IMRT (SIB-IMRT) (dashed line) for three patients. **(A)** Dose volume histogram (DVH) comparison in patient 1; **(B)** DVH comparison in patient 2; **(C)** DVH comparison in patient 3.

As shown in **Figure 4**, the dose received in PGTV and GTV from D95 to D2 are significantly increased in SIB-IMRT plans. But the OARs DVH of SIB-IMRT plans was roughly in line with that of Con-IMRT plans. There is also no significant difference in NT structure between two types of plan for each patient.

DVH indexes of each OAR are listed in **Tables 4**–**6** for further analysis. The rectum and bladder had slightly lower volumes at higher dose levels in SIB-IMRT plans. For the bladder of the three cases, V_30_, V_50_, D_1cc_, and D_2cc_ of SIB-IMRT plans are better than Con-IMRT plans. Although other bladder DVH indexes of Con-IMRT plans is a little bit better than that of SIB-IMRT plans, the difference is too small to be clinically significant. Similar results are seen in the rectum and the intestines. On the whole, most of OARs DVH parameters in SIB-IMRT plans were superior to that in Con-IMRT plans.

**Table 4:** Summary of dose volume histogram (DVH)-based analysis for the bladder of the three patients.

No.	Category	V_20_ (%)	V_30_ (%)	V_40_ (%)	V_50_ (%)	D_1cc_ (cGy)	D_2cc_ (cGy)
1	Con-IMRT	100	100	**96**	33	5,210	5,176
	SIB-IMRT	100	100	99	**31**	**5,192**	**5,152**
2	Con-IMRT	**61**	43	30	14	5,322	5,307
	SIB-IMRT	67	**42**	**28**	**13**	**5,314**	**5,246**
3	Con-IMRT	**94**	45	**14**	1	5,038	4,999
	SIB-IMRT	95	**44**	15	1	**5,021**	**4,966**

The better results are bolded.

**Table 5:** Summary of dose volume histogram (DVH)-based analysis for the rectum of the three patients.

No.	Category	V_20_ (%)	V_30_ (%)	V_40_ (%)	V_50_ (%)	D_1cc_ (cGy)	D_2cc_ (cGy)
1	Con-IMRT	100	100	85	29	5,243	5,193
	SIB-IMRT	100	100	**85**	**27**	**5,221**	**5,166**
2	Con-IMRT	0	0	0	0	1,613	1,585
	SIB-IMRT	0	0	0	0	**1,582**	**1,559**
3	Con-IMRT	0	0	0	0	543	532
	SIB-IMRT	0	0	0	0	**462**	**449**

**Table 6:** Summary of dose volume histogram (DVH)-based analysis for the intestines of the three patients.

No.	Category	V_20_ (%)	V_30_ (%)	V_40_ (%)	V_50_ (%)	D_1cc_ (cGy)	D_2cc_ (cGy)
1	Con-IMRT	10	4	1	0	4,590	4,449
	SIB-IMRT	10	**3**	1	0	**4,539**	**4,397**
2	Con-IMRT	64	39	22	7	**5,167**	**5,176**
	SIB-IMRT	**63**	**38**	**22**	7	5,192	5,206
3	Con-IMRT	**52**	33	17	3	5,130	5,051
	SIB-IMRT	53	**31**	**14**	**2**	**5,128**	**5,020**

### Follow-Up

**Figure 5:**
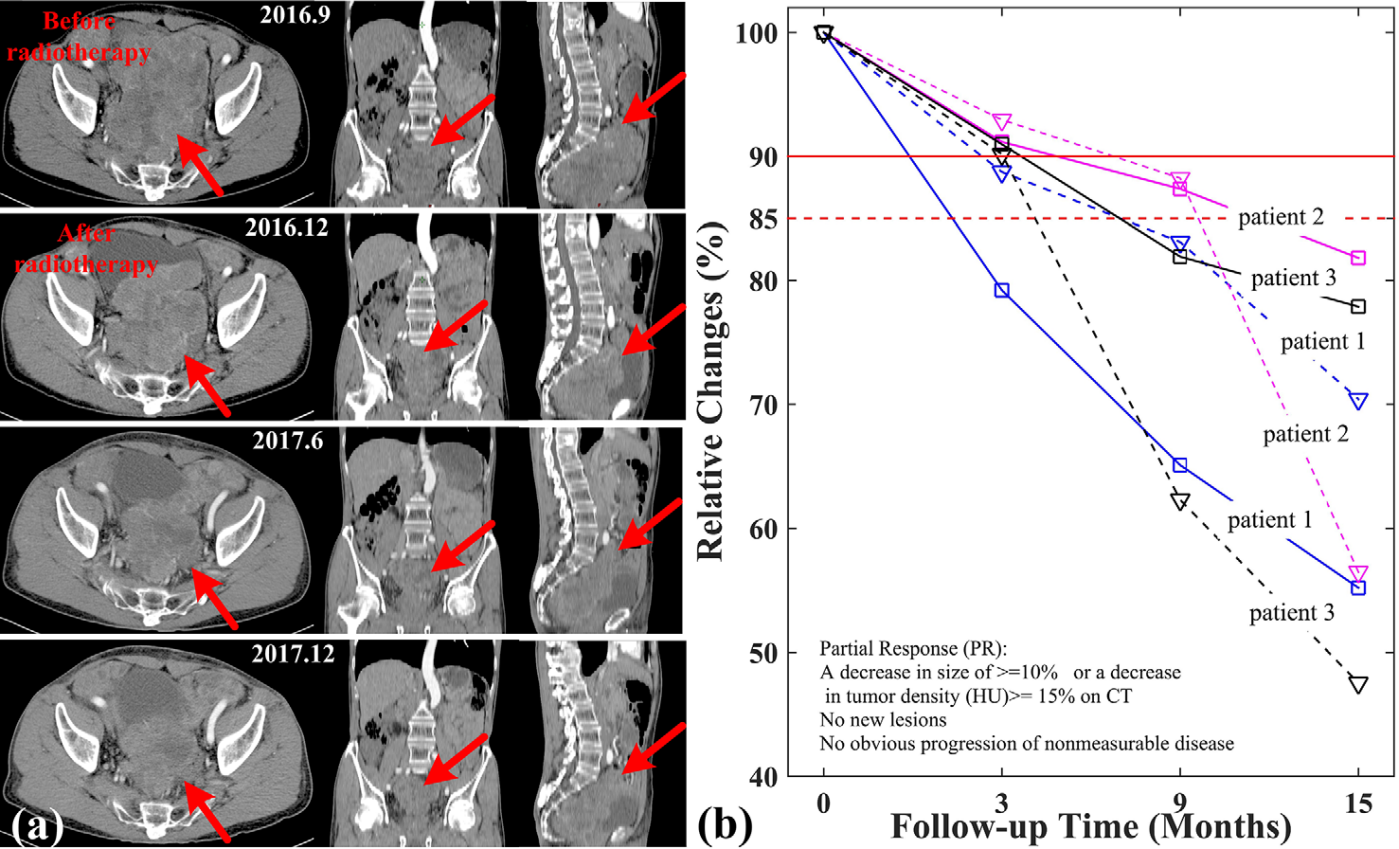
Relative changes of tumor size and CT values in three patients. **(A)** CT imaging of patient 1; **(B)** Relative changes of tumor size (solid line) and CT (dashed line) values in three patients.

During applying SIB-IMRT plans, three patients were well tolerated and their symptoms caused by abdominal mass compression were gradually alleviated. Their abdominal discomfort and deleterious effect disappeared after the end of treatment. As shown in **Figure 5A**, tumor lesion of patient 1 diminished obviously in follow-up CT examination. The gradually decreased size of tumor and CT values were observed in the CT imaging. The relative changes in tumor size and CT values of three patients was tracked in **Figure 5B**. Patient 2 and patient 3 also saw their irradiated lesions continuously shrinked within one year after treatment. More importantly, patient 1 had no tumor progression for nearly 2 years after radiotherapy. Based on the Choi criteria (27), the three patients were assessed as partial response (PR).

## Discussion

Rare intra-abdominal tumors, localized GISTs are typically treated with surgical resection. So, TKIs therapy is a recommended option for recurrent, metastatic or unresectable patients. However, it is well-known that about 40–50% of GISTs recurs after surgery. In addition, resistance to TKIs therapy is also a known clinical problem (28). The post-resistance treatment presents a huge challenge for the management of LADR-GISTs. Under this circumstance, radiotherapy may be a valuable alternative in LADR-GISTs with curative intent. In our study, three patients with LADR-GISTs were treated with SIB-IMRT plans respectively. Their irradiated lesions were generally in good control through the subsequent radiological examination. The results demonstrate that SIB-IMRT technique is feasible in LADR-GISTs and the role of radiotherapy in GISTs may have been underestimated (29, 30).

Historically, radiotherapy has been less commonly considered in GISTs due to two reasons: the moderate radiosensitivity in GISTs and the dose-limiting toxicity to adjacent intra-abdominal organs.

First of all, radiotherapy is mainly used for local control of abdominal metastases and relief of symptoms (25). Conventional fractionation and modest cumulative dose were recommended for GISTs. The total bioequivalent dose that was frequently used ranged from 30 to 50 Gy (31). In addition, a uniform dose distribution was commonly recommended within target volume and the maximum dose was limited within 110–115% to the prescription dose. However, in the prospective study of Joensuu et al. (32), only 2 out of 25 GIST patients achieved partial response under conventional radiotherapy. Moreover, the tumor was usually under control only for a few months (12, 33). In fact, GISTs are the commonest sarcoma in the gastrointestinal tract and relatively resistant to conventional dose schemes (26). Therefore, a higher biological equivalent dose are needed, especially in hypoxic area of tumor central region. Furthermore, an ablative does escalated to the subvolume of tumor has been proven to be more effective such as prostate, liver, or sarcoma as early as 1986 (34). Nomiya et al. (20) and Cilla et al. (17) demonstrated a heterogeneous dose distribution by SIB-IMRT technique could induce a higher rate of tumor cell apoptosis in bulky and hypoxic tumors, that were not controlled using. That cannot be achieved by conventional radiotherapy. In our study, the prescription dose of PGTV and GTV-outer was set to 50.4 Gy for a pathologic complete response (10). Meanwhile, the prescription dose of GTV-mid and GTV-center was boosted up to 60–62 Gy and 62–64 Gy respectively. Therefore, an ablative-like dose distribution was generated by SIB-IMRT and a steeper dose gradient within GTV was observed in **Figure 3**. Although the maximum dose in three patients were respectively escalated up to 129, 135, 131% of the prescribed dose in PGTV, only a slight increase of dose to NT structure were seen from profiles comparison. More importantly, the three patient were well-tolerated during the radiotherapy and continuous reduction in tumor size and CT values were found in the follow-up. The results about an ablative-like dose distribution by SIB-IMRT is feasible for large tumors. That was confirmed again in LADR-GISTs, which were historically considered to be relatively radio-resistant. Of course, the lesion reduction in patient 2 seemed to be less obvious than that in other patients. The reason may be that the area of patient 1 which received higher radiation dose was smallest of the three patients in **Figure 3**. It also implied that the proportion of area which received higher radiation dose had an impact on the tumor response. In addition, the SIB-IMRT plans required averagely 93 more MUs to be delivered compared with the Con-IMRT plans, under approximately identical practical treatment time. Above all, the results of our study imply that our SIB-IMRT plans has the potential to obtain an effective high tumor control with negligible treatment toxicities in the management of GISTs.

Secondly, the radiotoxicity of healthy tissue is another factor of concern in GISTs. For one thing, the gastrointestinal location, patterns spread and tumor size would potentially require large abdominal fields (6). For another, it is difficult to target tumor in a mobile segment of the gastrointestinal (31). So, radiotherapy may raise the risk in toxicities of the small bowel and visceral structures. Although IMRT has a significant reduction in acute and delayed toxicity of abdominal RT in recent years, large abdominal fields mean that it is still difficult to deliver too high radiation dose by Con-IMRT in bulky GISTs. SIB-IMRT may provide a means to deal with the dilemma of GISTs between increasing the radiation dose of target volume and alleviating radiotoxicity of OARs. In our study, GTV-mid and GTV-center was built on the contraction of GTV. Meanwhile, a higher radiation dose was delivered to the two parts to improve tumor’s response. A relaxed upper dose constraint was assigned for the GTV-outer and GTV-mid during optimization process in order to avoid higher radiation dose in the overlap region of OARs and GTV. As shown in **Tables 4**–**6**, a dose boost to target volume had no risk of overdosing the OARs. Some of OARs DVH indexes in SIB-IMRT plans were even lower than that in Con-IMRT plans. The reason may be that the relaxation of GTV upper dose constraint in SIB-IMRT plans increased the relative weight of all other constraints. Sun et al. (35) already involved in similar study. He concluded that removing the upper dose constraints in target volume may theoretically improve the OARs sparing and tumor control probability. In addition, the large tumor size of three patients also created a good condition for radiotherapy in our study. It is because that the relatively fixed tumor in the abdominal cavity is easily to be targeted. Nevertheless, rigorous IGRT is essential to the efficacy and safety of radiotherapy.

There are also some limitations in our study. Firstly, our sample size was small, only three patients, and the follow-up was short. That is why we reached the conclusion through observation and comparative analysis rather than statistical analysis. Secondly, despite the fact that some patients with LADR-GISTs have acquired efficacy through radiotherapy, further research is needed to make certain the optimal radiation dose schedule. Finally, although three patients did not take TKIs therapy after radiotherapy for personal reasons, radiotherapy influenced by TKIs therapy is a noticeable problem and will be an attractive topic. To sum up, it was just our preliminary study, and we will expand the sample size to continue our exploration in the future.

## Conclusion

A novel SIB-IMRT technique was designed for locally advanced drug-resistant GISTs and a heterogeneous dose distribution was escalated from the peripheral region of GTV to the center region. Compared to the Con-IMRT plans, the SIB-IMRT plans had the potential to improve the tumor response without significant increase in the radiotoxicity of the adjacent normal tissue in LADR-GISTs. Radiotherapy may be underutilized for GISTs, and SIB-IMRT technique may provide a new method for achieving objective response and prolonging survival in selected GISTs patients.

## Data availability statement

The raw data supporting the conclusions of this article will be made available by the authors, without undue reservation.

## Ethics statement

This study was reviewed and approved by the Ethical and Scientific Committees of the First Affiliated Hospital of Chongqing Medical University (Chongqing, China). The patient gave written informed consent in accordance with the Declaration of Helsinki. Written, informed consent was obtained from the patient individual(s) for the publication of any potentially identifiable images or data included in this article.

## Author Contributions

YL, LL, and XY: drafting of work, analysis and interpretation of trials and literature, drafting of manuscript, and manuscript review. HC and XZ collected the data, reviewed the literature, and wrote the paper. JD and QJ prepared the figure and contributed in the revision of the literature. All authors contributed to the article and approved the submitted version.

## Funding

This work is funded by the Beijing Medical and Health Public Welfare Foundation “Medical Science Research Fund” project (No.: YWJKJJHKYJJ-B184084-Q14) and the Chongqing Yuzhong District Science and Technology Commission key project (No.20190101).

## Conflict of Interest

The authors declare that the research was conducted in the absence of any commercial or financial relationships that could be construed as a potential conflict of interest.
